# Quo Vadis after AEGIS: New Opportunities for Therapies Targeted at Reverse Cholesterol Transport?

**DOI:** 10.1007/s11883-025-01281-3

**Published:** 2025-02-26

**Authors:** Nick S. R. Lan, Gerald F. Watts

**Affiliations:** 1 Medical School, The University of Western Australia, Crawley, Perth, Australia; 2 Department of Cardiology, Fiona Stanley Hospital, Perth, Australia; 3 Departments of Internal Medicine and Cardiology, Royal Perth Hospital, Perth, Australia

**Keywords:** Cardiovascular diseases, Coronary artery disease, Dyslipidaemia, Lipid-lowering therapy, Risk factors, Cholesterol

## Abstract

**Purpose of Review:**

High-density lipoprotein (HDL) is integral to reverse cholesterol transport (RCT), a process considered to protect against atherosclerotic cardiovascular disease (ASCVD). We summarise findings from the recent AEGIS-II trial and discuss new opportunities for HDL therapeutics targeted at RCT.

**Recent Findings:**

Mendelian randomisation studies have suggested a causal association between the functional properties of HDL and ASCVD. However, the AEGIS-II trial of CSL112, an apolipoprotein A-I therapy that enhances cholesterol efflux, did not meet its primary endpoint. Exploratory analyses demonstrated that CSL112 significantly reduced ASCVD events among participants with a baseline low-density lipoprotein (LDL)-cholesterol ≥ 100 mg/dL, suggesting that RCT may depend on LDL-cholesterol levels.

**Summary:**

The role of HDL therapeutics in patients with familial hypercholesterolaemia, inherited low HDL-cholesterol and impaired HDL function, especially with inadequately controlled LDL-cholesterol, merits further investigation. The treatment of patients with monogenic defects in HDL metabolism remains a significant gap in care that needs further research.

## Introduction

Atherosclerotic cardiovascular disease (ASCVD) remains a major cause of morbidity and mortality worldwide despite major advances in care [1]. High-density lipoprotein (HDL) has long been recognised as having a critical role in reverse cholesterol transport and protection against ASCVD [2]. An inverse relationship between plasma HDL-cholesterol levels and the risk of ASCVD was demonstrated in the 1960–70’s [3–5]. However, randomised clinical trials of therapies that can increase HDL-cholesterol levels, such as cholesteryl ester transfer protein (CETP) inhibitors, niacin and fibrates, have not shown significant reductions in ASCVD events [6–12]. Mendelian randomisation studies have suggested that levels of HDL-cholesterol and apolipoprotein A-I (apoA-I; the major apolipoprotein of HDLs) are not causally linked with ASCVD [13–16]. Consequently, interest in therapies that increase HDL-cholesterol levels has diminished. Moreover, accumulating evidence from observational studies suggests that the relationship between HDL-cholesterol levels and ASCVD may be “U-shaped”, with very high HDL-cholesterol levels being paradoxically associated with increased risk of ASCVD and all-cause mortality [17–19].

**Fig. 1:**
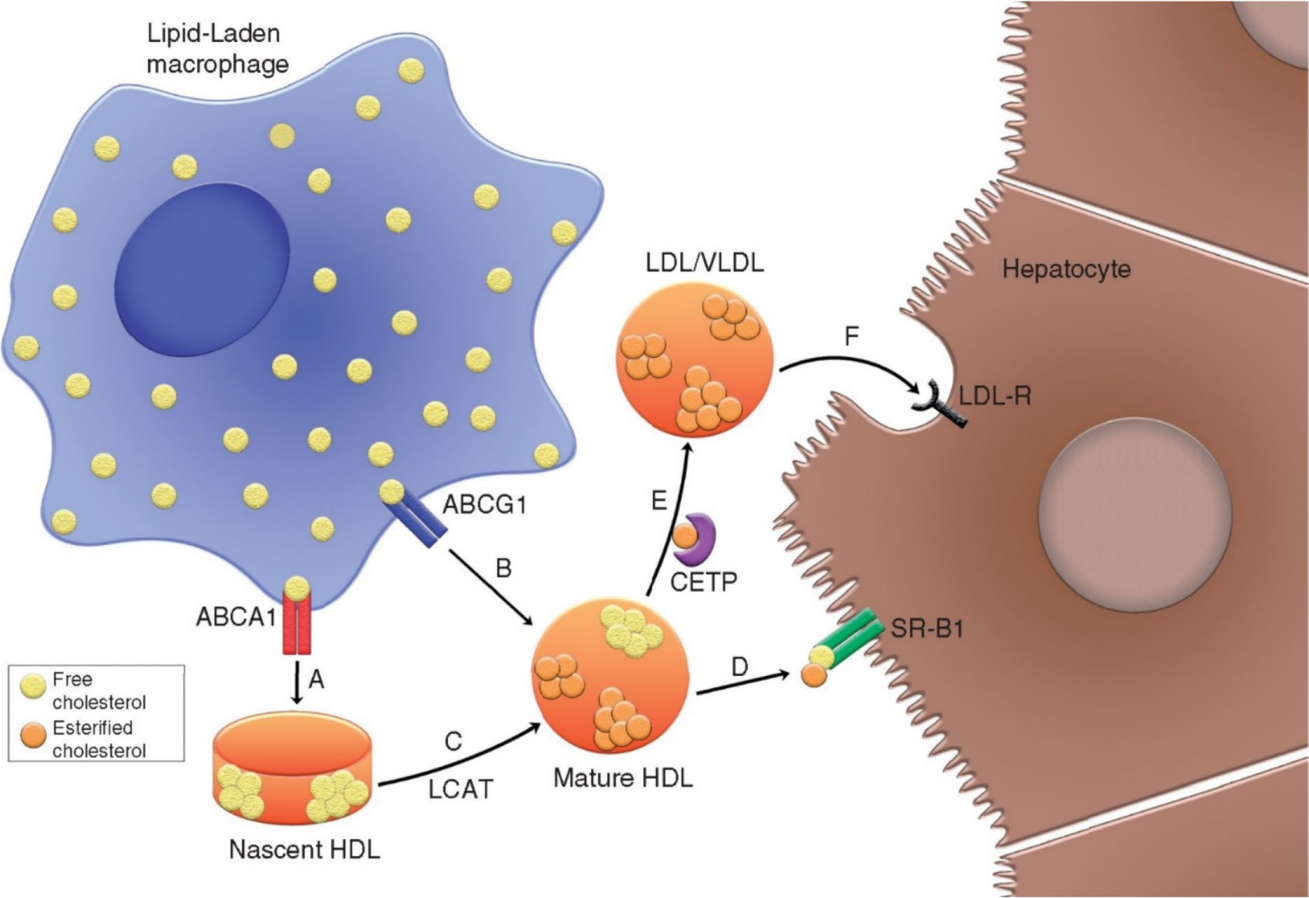
A simplified overview of reverse cholesterol transport [20]. Reproduced with permission from Oxford University Press (Allard-Ratick MP, et al. Eur J Prev Cardiol 2021).[20]. **A**) Free cholesterol is effluxed from lipid-laden macrophages (or foam cells) to nascent HDL particles in a process mediated by ABCA1. **B**) Free cholesterol is also effluxed from macrophages to mature HDL in a process mediated by ABCG1. **C**) LCAT esterifies free cholesterol to cholesteryl esters in nascent HDL, leading to the formation of mature HDL. **D**) Mature HDL is taken up by the liver via SR-B1 receptors. **E**) Cholesteryl esters are transferred from mature HDL to apoB-containing lipoproteins such as LDL and VLDL in a process mediated by CETP. **F**) LDL and VLDL are taken up by the liver via LDL receptors. Apolipoprotein A-I (not depicted) is the major structural protein of nascent and mature HDL particles. Abbreviations: ABCA1 ATP-binding cassette A1; ABCG1 ATP-binding cassette G1; apoB apolipoprotein B; CETP cholesteryl ester transfer protein; HDL high-density lipoprotein; LCAT lecithin:cholesterol acyltransferase; LDL low-density lipoprotein; SR-B1 scavenger receptor class B type 1; VLDL very low-density lipoprotein

Renewed interest in the therapeutic potential of targeting the HDL system has emerged owing to research on the functional properties of HDL. The most studied function of HDL is reverse cholesterol transport – the pathway whereby excess cholesterol is transported from peripheral tissues by plasma lipoproteins to the liver for excretion in the bile [20]. Reverse cholesterol transport (Fig. 1) begins with cellular cholesterol efflux, in which cholesterol is extracted from lipid-laden macrophages in a process mediated by apoA-I, ATP-binding cassette transporters (e.g., ABCA1 and ABCG1) and other proteins [2, 20]. HDL also possesses anti-inflammatory, anti-oxidative, anti-thrombotic and immunomodulatory properties that may protect against ASCVD [2, 21]. Importantly, plasma HDL-cholesterol concentrations do not reflect the function of HDL. Several studies have shown that cholesterol efflux capacity, HDL particle concentration, HDL particle size, HDL inflammatory index, HDL anti-inflammatory capacity and prebeta‐1 HDL level are better predictors of ASCVD than HDL-cholesterol concentrations [22–29]. However, the recent ApoA-I Event Reducing in Ischemic Syndromes II (AEGIS-II) trial demonstrated that CSL112, an apoA-I infusion therapy that enhances cellular cholesterol efflux, does not reduce ASCVD events when administered soon after an acute myocardial infarction (MI) [30].

In this review, we aimed to discuss: 1) recent Mendelian randomisation studies that support the causal role of functional aspects of HDL in ASCVD; 2) the AEGIS-II trial, including some of its limitations and new hypothesis-generating findings; and 3) further research avenues for HDL therapeutics, particularly in patients with monogenic disorders of LDL and HDL metabolism. The central objective was to develop after AEGIS the notion that there is still a case for testing the role of therapies directed at the HDL system, illustrating with examples of clinical contexts in which this would be most applicable. The complex structure, function and molecular regulation of HDL has been reviewed elsewhere, and a detailed discussion is beyond the scope of this review [2].

### Evidence from Mendelian Randomisation Studies

Mendelian randomisation studies provide a powerful tool to investigate possible causal relationships between risk factors or biomarkers and disease outcomes using analyses of the phenotypic consequences of specific genetic variations as the exposure (or intervention) of interest. The associations between the qualitative characteristics of HDL and ASCVD has only recently been tested using a Mendelian randomisation approach. The first Mendelian randomisation study on HDL qualitative traits and risk of ASCVD was performed by Prats-Uribe et al*.*, in which they suggested that there may be a causal link between genetically determined HDL characteristics and ASCVD [31]. The authors demonstrated that larger HDL diameter, higher cholesterol content in large HDL particles and higher triglyceride content in large HDL particles are associated with an increased risk of ASCVD [31]. On the other hand, higher cholesterol content in medium-sized HDL particles was inversely associated with the risk of ASCVD [31]. Another Mendelian randomisation study by Zhao et al*.*, also suggested a causal relationship between qualitative markers of HDL and ASCVD [32]. The authors demonstrated that the concentration and content of medium HDL particles, small HDL particles and smaller HDL particle diameter are associated with a protective effect against ASCVD [32]. Similar to the study by Prats-Uribe et al*.*, larger HDL particle diameter was found to be associated with a greater risk of ASCVD [32]. Importantly, consistent with earlier Mendelian randomisation studies, both the recent studies demonstrated that genetically determined HDL-cholesterol and apoA-I concentrations were not causally linked to ASCVD [31, 32].

### Implications for Previous Trials Targeting HDL

The findings of these recent Mendelian randomisation studies may explain why clinical trials have not shown significant reductions in ASCVD events with therapies that specifically increase HDL-cholesterol concentrations, without significantly altering the distribution of HDL particles and their properties [6–10]. First, niacin and CETP inhibitors can increase the level of large HDL particles [33, 34]. Second, niacin can increase the levels of atherogenic HDL proteins such as clusterin, haptoglobin/haptoglobin-related proteins and phospholipid transfer protein in statin-treated patients [35]. Third, CETP inhibitors can increase HDL particles that are enriched in apolipoprotein C-III and other subspecies that are associated with an increased risk of ASCVD [36]. Fourth, CETP inhibitors may reduce the hepatic clearance of HDL, potentially impairing reverse cholesterol transport, including faecal sterol excretion [37, 38]. Furthermore, participants in the relevant clinical trials were not selected for having an adverse qualitative HDL characteristics and functionality. Finally, it should be noted that Prats-Uribe et al*.*, and Zhao et al*.*, did not undertake instrumental variable analyses using cellular cholesterol efflux[31, 32], a key step in reverse cholesterol transport, identifying void in genetic data supporting the intervention used in the AEGIS-II trial.

### HDL Therapeutics Targeted at Reverse Cholesterol Transport

We now focus on HDL therapeutics targeted at reverse cholesterol transport and group them according to their predominant effects on the pathway (Table 1): 1) increasing the number and functionality of acceptors for cellular cholesterol efflux; 2) enhancing macrophage cellular cholesterol efflux; and 3) enhancing cholesterol esterification [39–41]. Importantly, the mechanistic role of reverse cholesterol transport in the protection against ASCVD has been demonstrated in several animal studies [42]. In humans, apoA-I-based therapies have been the most studied for their ability to modulate reverse cholesterol transport; several agents have been tested including apoA-I Milano, MDCO-216, CER-001, CSL111 and CSL112 [43]. However, beneficial effects on remodelling of atherosclerotic plaque have not been consistently confirmed [44–50]. More recently, studies have demonstrated that CSL112 can increase apoA-I levels and ABCA1-dependent cholesterol efflux more so than prior agents [43]. Unlike prior apoA-I-based therapies, CSL112 is a purified wild-type apoA-I that is derived from human plasma [43]. CSL112 can increase apoA-I levels by ~ twofold and cholesterol efflux capacity by ~ fourfold from baseline [30, 51]. CSL112 also accelerates the rate of free cholesterol esterification by LCAT, leading to HDL maturation [43]. Furthermore, apoA-I-based infusions have been shown to promote faecal excretion of cholesterol [52]. The biological properties and proposed mechanisms of CSL112 have been detailed elsewhere [43].

**Table 1:** Therapies classified according to mode of action in the early stages of reverse cholesterol transport pathway

Predominant action*	Therapy
Increase in number and function of acceptors for cellular cholesterol efflux	• ApoA-I infusions (e.g., apoA-I Milano, MDCO-216, CER-001, CSL111 and CSL112) • ApoA-I mimetic peptides • Up-regulators of endogenous apoA-I production • Autologous delipidated HDL
Stimulation of cellular cholesterol efflux	• Synthetic LXR agonists • Apolipoprotein E mimetics
Increase in rate of cholesterol esterification	• Recombinant LCAT • Small molecule activators of LCAT

Abbreviations: *ApoA-I* apolipoprotein A-I; *HDL* high-density lipoprotein; *LCAT* lecithin:cholesterol acyltransferase; *LXR* liver X receptor

^*^Therapies may affect multiple components of the reverse cholesterol transport pathway

### A Brief Overview of AEGIS-II

Cholesterol efflux capacity can be impaired following acute MI and is associated with subsequent ASCVD events and mortality [53, 54]. In the AEGIS-I trial (phase 2b), Gibson et al., confirmed the ability of CSL112 infusions to enhance cholesterol efflux and demonstrated its feasibility and safety in 1,258 patients with acute MI [51]. In the AEGIS-I trial, CSL112 was tested at both a low (2 g) and a high (6 g) dose, and dose-dependent elevations of apoA-I level and cholesterol efflux capacity were demonstrated [51]. Gibson et al*.*, then conducted the international, multi-centre AEGIS-II trial (phase 3) to determine whether enhancing cholesterol efflux with CSL112 can reduce ASCVD events in patients with acute MI [30]. The trial randomised 18,219 participants with acute MI, multivessel coronary artery disease and additional cardiovascular risk factors to weekly intravenous infusions of 6 g of CSL112 or matching placebo for 4 weeks [30]. The first infusion was administered within 5 days of medical review, but at least 12 h after the presentation with MI [30]. The primary endpoint was a composite of MI, stroke, or death from cardiovascular causes from randomisation through to 90 days of follow-up [30]. There was no significant difference between the groups in the primary endpoint (CSL112 4.8% versus placebo 5.2%; hazard ratio [HR] 0.93; 95% confidence interval [CI] 0.81–1.05; *P* = 0.24) [30]. CSL112 was generally well-tolerated; the number of patients who had anaphylactic or hypersensitivity reactions was low, but was higher in the CSL112 group (14 patients versus 4 patients; *P* = 0.02) [30].

### Limitations and Challenges of AEGIS-II

The AEGIS-II trial specifically evaluated a very high risk group of patients with acute MI in the early post-MI period. Hence, the findings are not directly applicable to other contexts, specifically to the care of patients with low HDL-cholesterol levels in chronic care settings. The trial had a very large sample size and 90% power for testing the effect of active treatment on the primary endpoint [30]. However, in patients with acute MI, the benefits of enhancing HDL function in reducing ASCVD events may be challenging to demonstrate on the background of intensive lowering of low-density lipoprotein (LDL)-cholesterol levels and secondary prevention therapies [30, 55]. Indeed, ~ 88% of patients underwent percutaneous coronary intervention and over 90% were treated with statin and anti-platelet therapies in the trial [30]. It is unclear whether a longer period of follow-up (i.e., > 1 year), earlier treatment (i.e., before, during or immediately after coronary intervention), or using a different dosing regimen (i.e., frequency, duration or dose) might have been more efficacious. As CSL112 has a volume of distribution and clearance rate similar to endogenous apoA-I (~ 48 h), it is possible that 4 infusions of the therapy over 4 weeks might have been insufficient to impact on established coronary plaques [43]. Notably, reduced cholesterol efflux capacity or impaired HDL functionality were neither inclusion criteria nor were such measurements reported before and after intervention. Selection of patients based on more complex measurements of HDL functionality would be scientifically interesting but feasible for clinical outcome trials [56].

### AEGIS Strikes Back: Exploratory Analyses and Generation of New Hypothesis

Insights into the potential benefits of CSL112 have been gained from exploratory analyses of the main AEGIS-II trial [55, 57, 58]. The authors found that participants treated with CSL112 had numerically lower rates of cardiovascular death and any type 1 MI compared with placebo at 90 days (HR 0.84; 95% CI 0.70–1.00; *P* = 0.056), 180 days (HR 0.86; 95% CI 0.74–0.99; *P* = 0.04) and 365 days (HR 0.89; 95% CI 0.79–1.01; *P* = 0.07) [57]. In another analysis, they found that CSL112 significantly reduced the total burden of nonfatal ischemic events and cardiovascular death compared with placebo at 180 days (745 versus 821 events; *P* = 0.04) and 365 days (1,120 versus 1,211 events; *P* = 0.04) [58]. Since type 2 MI is not thought to be modifiable by enhancing cholesterol efflux, the authors excluded it from the outcome of nonfatal MI or cardiovascular death and found a significant reduction in total ischaemic events at 90 days (342 versus 406 events; *P* = 0.02), 180 days (511 versus 606 events; *P* < 0.01) and 365 days (783 vs 881 events; *P* = 0.02) [58]. These analyses suggest that enhancing cholesterol efflux may reduce the lipid content of plaques and promote plaque remodelling into a more stable phenotype shortly after acute MI [57]. However, the effect of CSL112 on plaque composition merits further trials using imaging techniques. Such trials should consider selecting patients for having lipid-rich non-calcified plaque, which may be more responsive to the effects of CSL112. CSL112 also has anti-inflammatory and anti-oxidative properties, which may facilitate plaque stabilisation [43].

#### Potential Benefits According to Baseline LDL-Cholesterol

**Fig. 2:**
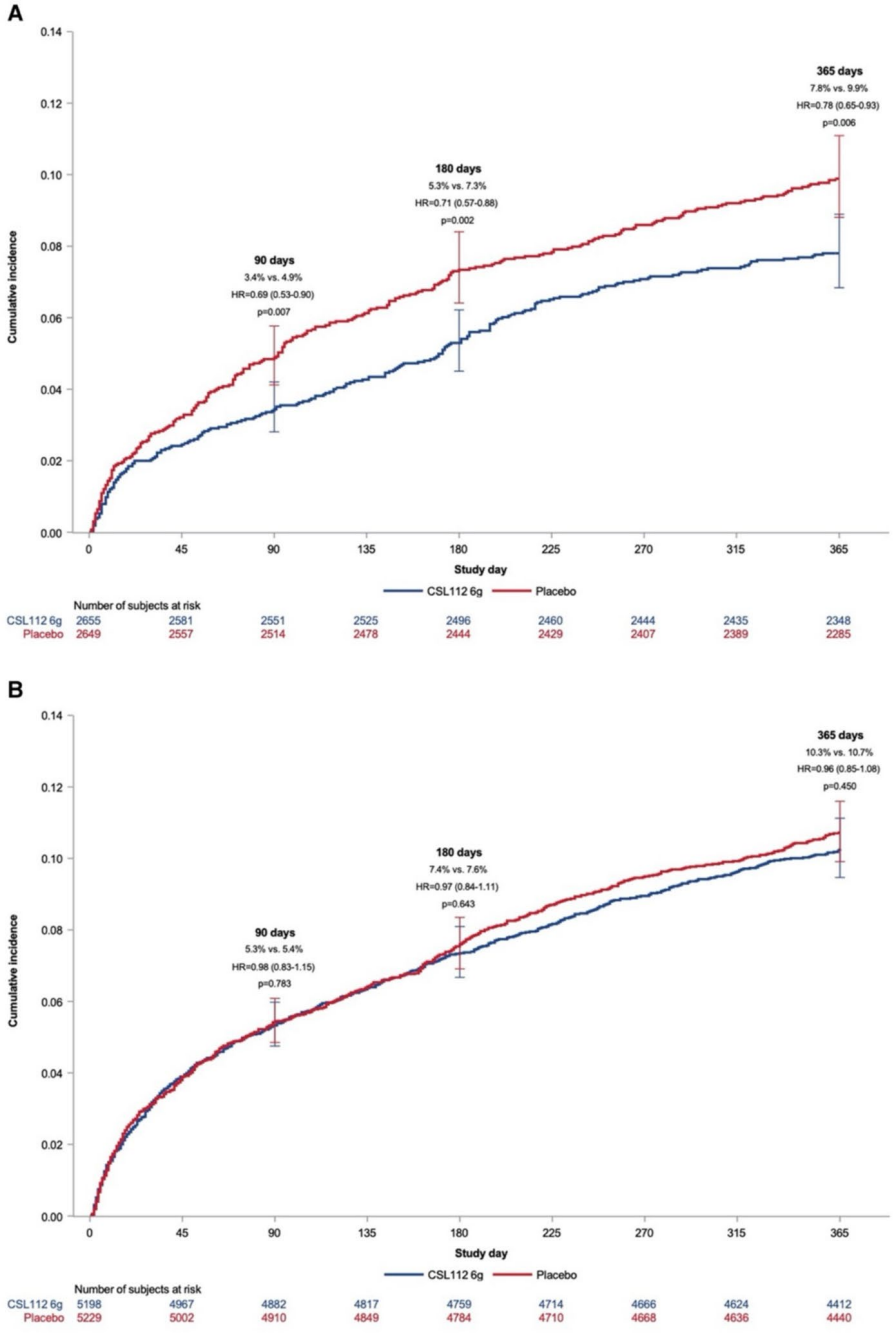
Cumulative incidence of time to first occurrence of the composite primary endpoint in the AEGIS-II trial at 90, 180 and 365 days [55]. Reproduced with permission from Oxford University Press (Gibson CM, et al. Eur Heart J 2024).[55]. **A**) Among patients with a baseline low-density lipoprotein cholesterol level ≥ 100 mg/dL who were prescribed statin therapy. **B**) Among patients with a baseline low-density lipoprotein cholesterol level < 100 mg/dL who were prescribed statin therapy

A particularly novel and important exploratory finding in the AEGIS-II trial was that treatment with CSL112 was associated with a significantly lower risk of cardiovascular events compared with placebo among participants with a baseline LDL-cholesterol level ≥ 100 mg/dL, but not among those with an LDL-cholesterol level < 100 mg/dL (Fig. 2) [55]. This suggests that patients who are hypercholesterolaemic may have a greater burden of lipid-rich plaque and therefore benefit more from CSL112 related to enhanced cholesterol efflux and other anti-atherosclerotic effects referred to above. However, it could also suggest that reverse cholesterol transport (Fig. 1) is dependent on the circulating concentrations of LDL. We have previously shown that cholesterol efflux capacity is directly dependent on the plasma concentration and kinetics of apoB100-containing lipoprotein particles [59]. In essence, a critical “pool size” of both LDL and VLDL particles may be necessary to enable direct cholesterol transfer from HDL particles and/or to accelerate the hetero-exchange of cholesterol between HDL and LDL/VLDL, with the net effect of maintaining a higher rate of reverse cholesterol transport to the liver [59]. This is supported by our recent data demonstrating that reductions in plasma apoB-100 concentration following lipid-lowering therapy correlate with reductions in cholesterol efflux capacity [60]. A recent mouse study has also demonstrated that LDL can induce cellular cholesterol efflux and that the LDL receptor can sustain reverse cholesterol transport [61].

### New Opportunities for Familial Hypercholesterolaemia and Lipoprotein(a)?

The aforementioned hypothesis, partially based on the exploratory analysis from the AEGIS-II trial [30], that reverse cholesterol transport is maintained by a higher plasma LDL pool size, supports further studies of HDL therapeutics in hypercholesteroleamic states. We speculate that certain patients with familial hypercholesterolaemia (FH), a monogenic disorder of LDL metabolism (principally due to variants in the *LDLR* gene) could benefit specifically from HDL therapeutics. Heterozygous FH (HeFH) is a common (~ 1:250) autosomal dominant disorder characterised by lifelong elevation of LDL-cholesterol level and premature ASCVD [62]. Several studies have demonstrated that low HDL-cholesterol level is associated with a higher risk of ASCVD in patients with HeFH [63–65]. This is further underscored by the inclusion of HDL-cholesterol in FH-specific risk scores for ASCVD [66, 67]. A recent study of patients with primary hypercholesterolaemia, which includes FH, demonstrated that mortality is lower in those with higher HDL-cholesterol levels [68]. Indeed, higher HDL-cholesterol levels and larger HDL particles are features of “resilient” FH [69, 70]. Resilient FH has also been associated with less atherogenic gene expression profile related to HDL metabolism (such as a higher expression of *ABCA1* and *ABCG1*) and immune responses (such a lower expression of *STAT2* and *STAT3*) [70, 71]. Alterations in the structure and function of HDL particles, leading to impaired reverse cholesterol transport, have been described in FH [72, 73]. Notably, there may be a lower transfer of unesterified cholesterol and a higher transfer of triglycerides and phospholipids to HDL particles from LDL particles [74]. In patients with FH, there is also increased catabolism of HDL-apoA-I which can lead to lower HDL-cholesterol levels [64, 75].

Based on the collective evidence, we propose that apoA-I-based therapies such as CSL112 be tested in patients with FH who have low HDL-cholesterol level or dysfunctional HDL particles, and sub-optimally controlled LDL-cholesterol level despite available therapies. Many patients with FH have LDL-cholesterol levels ≥ 100 mg/dL despite treatments [76]. A recent real-world study demonstrated that in patients with FH treated with proprotein convertase subtilisin/kexin type 9 (PCSK9) inhibitors, the mean LDL-cholesterol level at 24 months was 79.7 (45.9) mg/dL in those with HeFH and 95.1 (60.2) mg/dL in those with homozygous FH [76]. A phase 3 clinical trial of inclisiran demonstrated that ~ 65% of participants with HeFH receiving inclisiran attained an LDL-cholesterol level < 100 mg/dL at day 510 [77]. Moreover, homozygous FH is a rare (1:300,000) condition that manifests with LDL-cholesterol levels > 400 mg/dL and is associated with a very high risk of ASCVD [62]. Initial phase 2 studies of CER-001 in patients with homozygous FH support further evaluation of related HDL therapeutics in this cohort [78]. An example of a clinical trial could be to evaluate whether apoA-I-based infusions, administered immediately after lipoprotein apheresis, can mitigate plaque progression in patients with homozygous FH or severe HeFH. Such a trial would be pragmatic, given that apheresis is performed weekly or bi-weekly and requires vascular access.

Lipoprotein(a) [Lp(a)] is a largely genetically determined LDL-like particle, in which apolipoprotein(a) is covalently bound to apoB [79]. Accumulating evidence supports Lp(a) as a causal risk factor for ASCVD and calcific aortic valve stenosis [79]. Globally, ~ 20–30% of individuals have an elevated Lp(a) level (> 50 mg/dL or 125 nmol/L) [79]. Like LDL particles, Lp(a) may serve as an acceptor of cholesteryl esters from HDL particles [60]. We have previously shown that in individuals treated with a PCSK9 inhibitor, the reduction in plasma Lp(a) level was significantly correlated with the reduction in whole plasma cholesterol efflux capacity [60]. However, recent studies have also suggested that elevated Lp(a) levels may inhibit cholesterol efflux, including in patients with FH [80, 81]. Taken together, clinical trials of therapies that enhance cholesterol efflux in individuals with elevated Lp(a) and impaired cholesterol efflux would be of interest. In the interim, additional studies are required to better understand the interplay between Lp(a) and reverse cholesterol transport.

### Care of Individuals with Monogenic Defects in HDL Metabolism: an Unmet Need

Monogenic conditions associated with very low HDL-cholesterol level (principally due to variants in the *APOA1*, *ABCA1* or *LCAT* genes) or very high HDL-cholesterol level (principally due to variants in the *CETP*, *SCARB1* or *LIPC* genes) are rare and understudied [82, 83]. These monogenic defects result in impaired reverse cholesterol transport and have been associated with an increased risk of ASCVD [42, 84]. Variants in the *APOA1* gene for example, results in undetectable or very low apoA-I levels, and therefore fewer acceptors for cholesterol efflux [83]. Moreover, variants in the *ABCA1* gene lead to impaired cellular cholesterol efflux, and in the case of Tangier disease, the presence of only pre-β1 HDL.[83] However, there remains no specific treatments for individuals with monogenic defects in HDL [82]. Clinical trials of therapies that increase the number of acceptors and/or stimulate cellular cholesterol efflux in these patients are merited. CER-001 has been evaluated in small phase 2 and 3 studies of individuals with genetically determined very low HDL-cholesterol levels (*APOA1*, *ABCA1* or *LCAT* gene variants), but anti-atherosclerotic benefits have not been consistently shown [85, 86]. This may be due to reasons such as: 1) insufficient plaque at baseline to demonstrate significant benefits; 2) insufficient dose; 3) insufficient follow-up duration; or 4) difficulties with demonstrating benefits when LDL-cholesterol levels are reduced with statin therapy [86]. In individuals with familial LCAT deficiency, where the ability to esterify cholesterol in HDL is impaired, recombinant LCAT and small molecules enhancing LCAT activity may be future therapeutic options [87–89]. However, repeated intravenous infusions may not be feasible for many patients, underscoring the need to develop alternative formulations (e.g., oral or injectable).

There is also a need to develop high-quality international registries for individuals with monogenic defects in HDL [82]. This would facilitate sharing of educational, clinical and research information, including clinical trials. Such registries should include patient-reported experience measures and be linked to clinical outcomes. Continued research into the genetic regulation of HDL will pave the way for identifying novel therapeutic targets. The use of gene-based therapies in the treatment of hereditary lipid disorders is increasingly promising [62]. Indeed, gene silencing therapies such as antisense oligonucleotides and small interfering RNAs are in advanced stages of clinical trials for several conditions or are being utilised for lipid management in practice (e.g., inclisiran). Splice correction therapies may be a novel option for precision medicine in families with monogenic defects in HDL that should be studied [90]. Splice correction with antisense oligomers are being tested in individuals with FH and certain variants in the *LDLR* gene that involve splicing defects [90]. Recent reports have further highlighted the impact of splicing variants in the *ABCA1* gene [91, 92]. However, a limitation of this strategy is that tailored antisense oligomers need to be designed for each variant in different exons.

### Counterpoint: CETP Inhibition as HDL Therapy?

The development of several CETP inhibitors have been discontinued due to toxicity or futility in large clinical outcome trials [12]. However, obicetrapib is a potent and selective oral CETP inhibitor that increases HDL-cholesterol levels by 140–160%, reduces LDL-cholesterol levels by 40–50% and reduces Lp(a) levels by 50–60% [93–95]. Obicetrapib has recently progressed to a phase 3 ASCVD outcomes trial (PREVAIL: NCT05202509), which will enrol 9541 participants with ASCVD who are receiving maximally tolerated lipid-lowering therapy and have inadequately controlled LDL-cholesterol levels (≥ 55 mg/dL). Three other phase 3 trials are evaluating obicetrapib in participants with ASCVD, HeFH or at high risk for ASCVD (BROADWAY: NCT05142722, BROOKLYN: NCT05425745, TANDEM: NCT06005597). The results of BROOKLYN were presented at the American Heart Association 2024 Annual Scientific Sessions. It should be noted that the ASCVD benefit observed with prior CETP inhibitors is due to lowering of apoB-containing particles [i.e., LDL and Lp(a)] rather than to an increase in HDL-cholesterol concentrations [12, 96]. Intriguingly, the substantial increase in HDL-cholesterol with CETP inhibitors suggests that HDL-mediated reverse cholesterol transport is blocked. Essentially, cholesterol in HDL is not being removed by the liver from the circulation and is not being transferred to apoB-containing particles for transport back to the liver. It remains to be seen whether obicetrapib can reduce ASCVD events, and if so, whether this reduction is consistent with the full anticipated benefits of lowering apoB-containing particles or whether it is offset by impaired reverse cholesterol transport. However, it is important to note that *CETP* gene variants associated with high HDL-cholesterol levels and low LDL-cholesterol levels have been associated with a lower risk of ASCVD, supporting a causal role and value of CETP inhibitors in the reduction of clinical events [96–99].

## Conclusion

The role of targeting the “HDL pathway” for the prevention of ASCVD is supported by biological plausibility and recent genetic evidence but needs to be further tested in clinical trials. Although the overall results of the AEGIS-II trial was negative for its primary endpoint, exploratory analyses have provided important insights, suggesting that HDL therapies targeted at the initial steps of reverse cholesterol transport, particularly increasing the number and function of acceptors for cellular cholesterol efflux (Table 1), are worthy of further study. Notably, adequate levels of apoB-containing lipoproteins may be required to effectively maintain reverse cholesterol transport. This concept offers new opportunities for targeting HDL function in patients with FH and possibly elevated Lp(a). However, the optimal disease state, treatment regimen, timing of intervention and follow-up duration for trials of HDL therapeutics targeted at reverse cholesterol transport still needs to be identified. In the interim, the management of patients with very low HDL-cholesterol level is to address secondary causes and intensively manage traditional ASCVD risk factors, including modifying the risk associated with LDL-cholesterol and triglyceride-rich lipoproteins with currently available treatment strategies. In patients with obesity, incretin-based therapies for weight loss are a novel strategy to significantly reduce triglyceride levels and increase HDL-cholesterol levels [100]. Lastly, international registries and research into novel HDL therapeutics is needed to address the gap in the care of individuals and their families with monogenic defects in HDL.

## Key References

- Rohatgi A, Westerterp M, von Eckardstein A, Remaley A, Rye KA. HDL in the 21st Century: A Multifunctional Roadmap for Future HDL Research. Circulation. 2021;143(23):2293–309. 10.1161/circulationaha.120.044221.
  - A review on the structure and function of HDL.
- Gibson CM, Duffy D, Korjian S, Bahit MC, Chi G, Alexander JH, et al. Apolipoprotein A1 Infusions and Cardiovascular Outcomes after Acute Myocardial Infarction. N Engl J Med. 2024;390(17):1560–71. 10.1056/NEJMoa2400969.
  - The AEGIS-II trial publication.
- Povsic TJ, Korjian S, Bahit MC, Chi G, Duffy D, Alexander JH, et al. Effect of Reconstituted Human Apolipoprotein A-I on Recurrent Ischemic Events in Survivors of Acute MI. J Am Coll Cardiol. 2024;83(22):2163–74. 10.1016/j.jacc.2024.03.396.
  - Exploratory analysis of AEGIS-II showing a reduction in recurrent ischaemic events.
- Gibson CM, Chi G, Duffy D, Bahit MC, White H, Korjian S, et al. ApoA-I Infusions and Burden of Ischemic Events After Acute Myocardial Infarction: Insights From the AEGIS-II Trial. J Am Coll Cardiol. 2024;84(22):2185–92. 10.1016/j.jacc.2024.08.001.
  - Exploratory analysis of AEGIS-II showing a reduction in total ischaemic events.
- Gibson CM, Duffy D, Bahit MC, Chi G, White H, Korjian S, et al. Apolipoprotein A-I infusions and cardiovascular outcomes in acute myocardial infarction according to baseline LDL-cholesterol levels: the AEGIS-II trial. Eur Heart J. 2024;45(47):5023–38. 10.1093/eurheartj/ehae614.
  - Exploratory analysis of AEGIS-II showing benefit of CSL112 in participants with LDL-cholesterol ≥ 100 mg/dL.
- Korjian S, Kazmi SHA, Chi G, Kalayci A, Lee JJ, Talib U, et al. Biological basis and proposed mechanism of action of CSL112 (apolipoprotein A-I [human]) for prevention of major adverse cardiovascular events in patients with myocardial infarction. Eur Heart J Cardiovasc Pharmacother. 2023;9(4):387–98. 10.1093/ehjcvp/pvad014.
  - A review on the mechanisms of action of CSL112.
- Ying Q, Ronca A, Chan DC, Pang J, Favari E, Watts GF. Effect of a PCSK9 inhibitor and a statin on cholesterol efflux capacity: A limitation of current cholesterol-lowering treatments? Eur J Clin Invest. 2022;52(7):e13766. 10.1111/eci.13766.
  - A study supporting the hypothesis that reverse cholesterol transport is maintained by a higher plasma LDL [and possibly Lp(a)] pool size.
- Tamehri Zadeh SS, Chan DC, Mata P, Watts GF. Coronary artery event-free or resilient familial hypercholesterolemia: what's in a name? Curr Opin Endocrinol Diabetes Obes. 2024. 10.1097/med.0000000000000874.
  - A review that includes a discussion on HDL in patients with familial hypercholesterolaemia.
- Nurmohamed NS, Ditmarsch M, Kastelein JJP. Cholesteryl ester transfer protein inhibitors: from high-density lipoprotein cholesterol to low-density lipoprotein cholesterol lowering agents? Cardiovasc Res. 2022;118(14):2919–31. 10.1093/cvr/cvab350.
  - A review on CETP inhibitors and their potential cardiovascular benefits.

## Data Availability

No datasets were generated or analysed during the current study.
